# Effect of Different Aqueous Solvents with and Without Solubilized Lignin on the Swelling Behavior of Holocellulose Fibers

**DOI:** 10.3390/polym17233103

**Published:** 2025-11-22

**Authors:** Cornelia Hofbauer, Thomas Harter, Ulrich Hirn, Michael Harasek, Luis Zelaya-Lainez, Josef Füssl, Markus Lukacevic, Sebastian Serna-Loaiza

**Affiliations:** 1 Christian Doppler Laboratory for Next-Generation Wood-Based Biocomposite, Institute of Chemical, Environmental and Bioscience Engineering, TU Wien, 1060 Vienna, Austria; harter@tugraz.at (T.H.); michael.harasek@tuwien.ac.at (M.H.); sebastian.serna@tuwien.ac.at (S.S.-L.); 2 Institute of Bioproducts and Paper Technology, TU Graz, 8010 Graz, Austria; ulrich.hirn@tugraz.at; 3 Christian Doppler Laboratory for Next-Generation Wood-Based Biocomposite, Institute for Mechanics of Materials and Structures, TU Wien, 1040 Vienna, Austria; luis.zelaya@tuwien.ac.at (L.Z.-L.); josef.fuessl@tuwien.ac.at (J.F.); markus.lukacevic@tuwien.ac.at (M.L.)

**Keywords:** holocellulose, impregnation, spruce wood, swelling

## Abstract

The modification of lignocellulosic fibers through controlled swelling and impregnation plays a decisive role in tailoring their structure and reactivity for use in sustainable composite materials. In this study, holocellulose fibers were swollen in various solvents (sodium hydroxide at 2 and 4 wt% and ethanol–water mixtures at 0, 50, 70, and 100 wt%) to evaluate their impact on swelling and fiber characteristics. The pulp was produced with peracetic acid at 90 °C for 120 min from spruce wood chips and used for the swelling treatment. The fibers underwent swelling for 4 h in the different solvents, both without and with solubilized lignin at concentrations of 10 and 30 g/L, to investigate the impregnation ability of the fibers for lignin as a natural binder. Fiber morphology, lignocellulosic composition, and liquid retention values were analyzed to assess the effects of solvent–binder interactions on fiber swelling and lignin uptake. The results revealed significant differences in fiber characteristics influenced by both solvent choice and lignin presence, demonstrating the feasibility and optimization potential of a single-step swelling-impregnation process. These findings highlight key factors that can improve the uptake of natural binders in wood fibers, offering insights for effective fiber preconditioning in composite production.

## 1. Introduction

According to the Global ABC Report (2022) [1], the construction sector accounts for 37% of global CO_2_ emissions, 34% of total energy consumption, and nearly half of all resource use, while generating about 40% of global waste. These impacts are expected to intensify with growing construction demand, particularly in the global south. Wood-based construction offers a promising pathway to reduce emissions through long-term carbon storage and cascading material use. However, limited forest resources require innovative, circular solutions along the wood value chain. Establishing a bioeconomy based on renewable resources is therefore essential, emphasizing efficient biomass utilization and sustainable composite development [2]. In this context, wood-industry by-products such as sawdust, shavings, and wood chips represent valuable feedstocks containing intact fibers. Modifying these natural fibers and employing bio-based binders can replace fossil-based adhesives, contributing to low-carbon, high-performance materials for sustainable construction.

One promising application is the development of wood-fiber-based composites, offering a material use of the biomass. All-wood composites, which use only native components like cellulose, hemicellulose, and lignin, are a promising alternative to conventional composites such as medium-density fiberboard (MDF), which rely on synthetic and often hazardous adhesives. However, the challenge lies in developing sustainable processes to harness the potential of these fibers. There are already some promising approaches in research on transforming wood into biocomposites using only its natural components, without the need for synthetic adhesives or binders. For instance, [3] studied the mechanical and physical properties of hot-molded poplar wood powder without any additional binding agents and found promising results that compete with traditional medium- and high-density fiberboards.

Building upon these findings, it is important to note that cellulose fibers in wood, which serve as reinforcing elements, are naturally bonded by hemicellulose and lignin, contributing to strong binding within native wood. Therefore, using native wood’s structure as a model for creating a sustainable biocomposite, it becomes crucial to retain hemicellulose during fiber extraction [4,5]. Environmentally friendly methods, such as peracetic acid (PAA) pulping [6], are increasingly favored as they produce hemicellulose-rich pulp (holocellulose). This pulping method is highly selective towards delignification, while retaining a vast amount of hemicellulose [7]. PAA pulping results in long, intact fibers with moderate mechanical treatment (stirring), preserving their natural properties [8]. Westin et al. demonstrated that an optimized one-step PAA process yields holocellulose fibers with well-maintained hemicellulose and cellulose content at approximately 60% yield and good mechanical properties [9]. Due to their high fiber length, low residual lignin, and preserved polysaccharide content, holocellulose fibers represent an ideal reinforcement material for investigating binder interactions in biocomposites.

A binder can also be obtained from the same source as the fiber material [10]. Lignin, a naturally abundant polymer, has the potential to function as a binder due to its compatibility with cellulose and hydrophobic characteristics, which enhance composite strength, durability, and moisture resistance. Jiang et al. [11] demonstrated the advantages of lignin as a binding material for cellulose, improving the tensile strength and water resistance of a fiber network due to its hydrophobic behavior. Gouveia et al. tested Kraft lignin as a binder for eucalyptus fibers to produce medium-density fiberboards (MDF), which met the requirements of European standards for both thickness swell (TS) and internal bonding (IB) for indoor applications [12]. However, lignin distribution within composites strongly depends on its dissolution and precipitation dynamics in the swelling solvent. To further enhance lignin–fiber interactions, there are two ways to improve the bonding. One is the structuration of the lignin to enhance its adhesive performance, which is closely linked to its molecular structure and morphology. A uniform molecular arrangement and balanced hydroxyl group distribution enhance bonding with lignocellulosic polymers [13]. Moreover, lignin can self-assemble into colloidal spheres under solvent–antisolvent conditions, forming auto-adhesive structures that strengthen composite interfaces and increase tensile strength by up to 50% [14]. On the other hand, both components can be chemically activated through treatments such as peroxide [15,16,17], TEMPO [18,19], Fenton oxidation [20], or by functionalization strategies like carboxymethylation and maleation [21,22]. Recent advances in physical and chemical lignin modification techniques, including ultrasonic treatment and hydroxymethylation [23], have further improved its cross-linking ability and binding performance in sustainable adhesive applications. These modifications introduce reactive sites that facilitate covalent bonding during hot pressing, thereby enhancing water resistance and overall composite performance. Moreover, the presence of hemicelluloses can promote the formation of hydroxymethylfurfural (HMF) under heat, contributing to additional crosslinking and improved structural integrity of the composite [24].

An essential step in modifying the fibers is swelling, which can significantly influence their ability to interact with binders. This characteristic is based on increased fiber flexibility and reactive surface area [25], facilitating better adhesion to binders and other fibers due to the increased softness of the surface [26,27]. Various swelling agents can increase surface availability and improve bonding mechanisms, while also acting as binder carriers, thereby simplifying composite manufacturing processes [28,29].

Many suitable swelling agents are known for their ability to swell natural fibers to tune different surface properties [30]. Water, the most common swelling agent, safely swells fibers without causing structural damage, but exhibits limited capacity to dissolve hydrophobic binders like lignin [31]. Sodium hydroxide stands out as an effective swelling agent, evenly swelling fibers and efficiently carrying binder materials into the fiber structure. However, sodium hydroxide treatments may partially dissolve polysaccharides, potentially weakening the mechanical properties of fibers [32,33]. Environmentally friendly organic solvents, such as ethanol–water mixtures, also represent promising alternatives. Pure ethanol has limited swelling capabilities but effectively dissolves hydrophobic binders. When mixed with water, ethanol solutions offer additional advantages, as water can enhance the solubility of the binder [34] or act as an anti-solvent, facilitating lignin precipitation [35]. This dual behavior offers a significant advantage for tuning impregnation strategies in bio-based composites. The challenge lies in finding the right balance between water and ethanol and the timing for introducing the water into the system.

While previous research has explored various swelling solvents independently, little is known about how their interaction with binder materials, particularly lignin, affects fiber swelling and binder impregnation efficiency. Understanding these interactions is crucial, as they influence not only the composite’s structural integrity but also the practicality of combining swelling and impregnation into a streamlined, single-step process.

To address the identified research gap, this study is structured in two parts to investigate the behavior of spruce holocellulose fibers when treated with different solvent systems. Part I investigates the effects of different swelling agents on holocellulose fibers, using sodium hydroxide solutions (2 wt% and 4 wt%) and ethanol–water mixtures ranging from 0% to 100% ethanol. Part II evaluates whether the same solvents can simultaneously transport lignin into the fibers, thereby linking swelling behavior with impregnation efficiency in a single integrated process. Instead of treating swelling and impregnation as separate steps, this integrated approach reflects the core objective: to evaluate whether simultaneous swelling and lignin uptake is feasible and to identify solvent–binder systems that maximize impregnation efficiency and support simplified, sustainable composite manufacturing.

## 2. Materials and Methods

### 2.1. Materials

Norway spruce wood (*Picea abies*) sawmill chips were supplied by HS Timber Group in Kodersdorf, Germany, in various particle sizes (0.05 mm to 4 cm). Since the chips comprise sapwood, heartwood, juvenile wood, and knots in different fractions, their fiber dimensions naturally vary [36]. They were stored in plastic bags at room temperature with a moisture content of about 10 wt%. Structural carbohydrates and lignin of the wood chips were characterized according to NREL/TP-510-42618 [37], and ash content according to NREL/TP-510-42622 [38]. For the PAA process, 99.8% acetic acid (Sigma-Aldrich, Darmstadt, Germany) and 35% hydrogen peroxide (Donauchem, Vienna, Austria) were used. The chemicals used for swelling and lignin solutions included ethanol (96%, Australco, Spillern, Austria), deionized water, and sodium hydroxide pellets (Fischer Scientific, Geel, Belgium). The lignins used were Kraft lignin from softwood (UPM BioPivaTM 100, UPM Biochemicals, Leuna, Germany) with the specifics of molecular weight (Mw) = 2140 Da, total phenolics content (total-OH) = 3.5 mmol/g, and Organosolv lignin from hardwood (Fraunhofer CBP, Leuna, Germany) with the specifications of Mw = 1681 Da, total-OH = 2.5 mmol/g. The moisture content was 34.16 wt% for Kraft lignin and 4.49 wt% for Organosolv lignin.

### 2.2. Methods

#### 2.2.1. Experimental Plan

**Figure 1:**
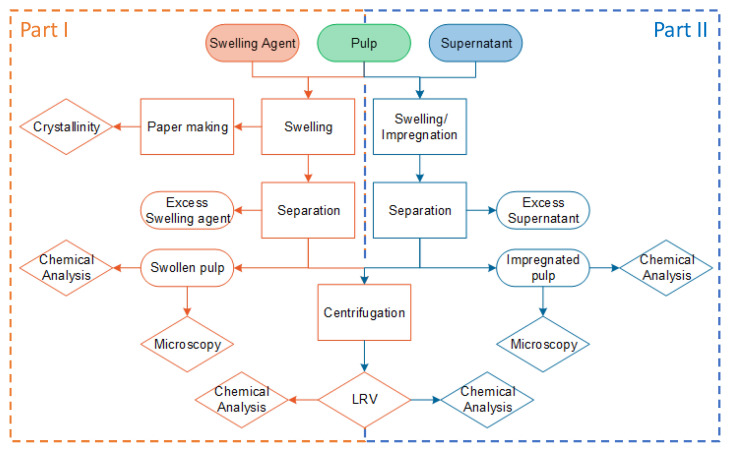
The study is divided into two parts. I: Solvent swelling process (orange), and II: the combined impregnation and swelling (blue). (LRV-Liquid Retention Value).

The key goals of the experimental plan are (1) to evaluate the fiber swelling ability of different solvents and (2) to evaluate the solvents’ ability to transport the dissolved lignin into the fibers, thus achieving an efficient impregnation of the fibers with lignin. Figure 1 shows an overview of the two preparation paths for swelling and swelling with impregnation, with the methods used to characterize the final product (crystallinity, microscopy, and LRV—Liquid retention value).

All swelling and impregnation experiments were performed in triplicate (except the LRV), and the results are presented as mean ± standard deviation. The low error margins (<5%) confirm the reproducibility and reliability of the obtained data.

#### 2.2.2. Preparation of Holocellulose Pulp

**Figure 2:**
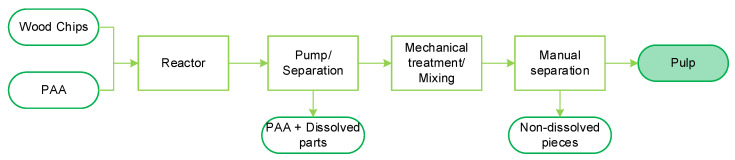
PAA-pulping process (holocellulose).

Holocellulose was prepared from spruce wood chips through pretreatment with peracetic acid (PAA), as shown in Figure 2. Initially, the wood chips were processed in a glass reactor containing a solution of acetic acid (99.9%) and hydrogen peroxide (35%) mixed in a 1:1 weight ratio. The wood chips were added to this PAA solution at a solid load of 3 wt% and heated to 90 °C for 120 min. During the final 60 min of heating and throughout the cooling phase down to 30 °C, the mixture was gently stirred with an impeller at 700 rpm to enhance defibrillation. After the reaction, the pulp was filtered through a nylon filter to separate it from the solvent and then washed with water until the pH reached ~6. The wet pulp was immersed in 15 L of water and mechanically stirred at 1700 rpm for 20 min, followed by an additional 5-min treatment with a hand mixer to break up most of the remaining fiber bundles. Finally, the pulp was separated from the water using the nylon filter and stored at 8 °C with a moisture content of 85–95 wt%. The pulping parameters (90 °C, 120 min) were selected based on previous experiments to ensure complete dissemination into single fibers while providing selective delignification and maintaining fiber morphology. This is critical for preserving fiber integrity within a homogenous pulp for subsequent swelling treatments.

Two independent batches of PAA-treated holocellulose were prepared using identical chemical charge and reaction conditions. The resulting pulps showed highly consistent composition (hemicellulose 32.4 ± 0.5 wt%; residual lignin 0.6 ± 0.1 wt%), confirming the reproducibility of the treatment.

#### 2.2.3. Swelling (Different Aqueous Solutions)

For the swelling, 6 different aqueous solutions were selected: 49.9 wt% aqueous ethanol (50% ethanol), 69.9 wt% aqueous ethanol (70% ethanol), 99.8 wt% aqueous ethanol (100% ethanol), 2 wt% aqueous NaOH, 4 wt% aqueous NaOH, and deionized water. The holocellulose pulp was first processed in a 1-L bucket to separate the pulp before introducing the fibers for swelling in the solutions for 4 h at room temperature. Although fiber swelling can continue over longer durations [39], the selected 4 h treatment time follows the procedures specified in ISO 23714:2014 [40] and ISO 5263-1 [41] to ensure methodological consistency.

#### 2.2.4. Lignin Solutions (Supernatant)

**Figure 3:**
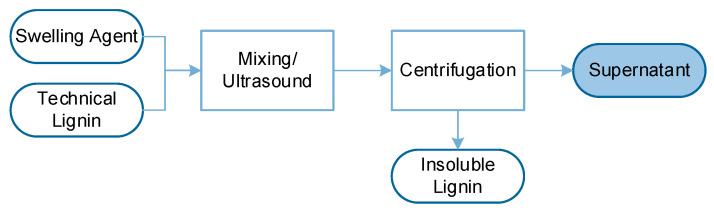
Process to produce lignin solution (supernatant).

The lignin supernatants were prepared with different swelling solutions and two types of lignin: from the Kraft (K) and Organosolv (OS) processes, as illustrated in Figure 3. Therefore, the commercial lignin powders were introduced into the different swelling solutions and mixed with a magnetic stirrer for 30 min. The dispersions were then ultrasonicated in a bath at 45 kHz and 200 W for 60 min (VWR, USC 2100 T, VWR Avantor, Radnor, PA, USA) while ensuring the temperature remained below 40 °C. This treatment was selected to improve dispersion without chemically altering the lignin. The effect of ultrasonication on lignin structure was verified by measuring the Mw before and after the ultrasonication, which showed no significant difference. Then, the dispersions were centrifuged at 5100 rpm for 20 min to separate the solubilized lignin solution (i.e., the supernatant) from the insoluble fraction. Each supernatant contained either 10 g/L (K10 and OS10) or 30 g/L (K30 and OS30) of solubilized lignin fraction. Based on preliminary solubility and viscosity tests, these concentrations were selected as they yielded stable lignin dispersions without visible precipitation and ensured reproducible impregnation behavior across treatments.

#### 2.2.5. Swelling with Impregnation

Prior to impregnation of the holocellulose fibers, the supernatant was tested for its actual gravimetric lignin concentration and diluted to the specific concentration of 10 or 30 g/L. Two grams of holocellulose fibers were introduced to 75 mL of supernatant and further processed according to the swelling protocol for the different aqueous solutions.

#### 2.2.6. Chemical Characterization

Holocellulose fibers were characterized using the same methods as those employed for analyzing the initial composition of the wood chips according to NREL/TP-510-42618 and NREL/TP-510-42622 [37,38]. Extractives were removed prior to hydrolysis using Soxhlet extraction (NREL/TP-510-42619) [42] to gravimetrically determine the content of extractives and discard significant interferences with the chemical characterization. Carbohydrates were quantified using the standardized two-step sulfuric acid hydrolysis procedure. In the first stage, samples were treated with 72% H_2_SO_4_ at ambient temperature to swell the biomass and initiate partial hydrolysis of polysaccharides. Subsequently, the acid concentration was diluted to 4%, and samples were autoclaved at 121 °C for 1 h to complete hydrolysis. The resulting monomeric sugars were analyzed using ultra-high-performance liquid chromatography (UHPLC; Thermo Scientific UltiMate 3000, equipped with a variable wavelength detector). A sugar recovery standard was included to correct for losses due to sugar degradation. These methods were also applied after the swelling and impregnation processes.

#### 2.2.7. Light Microscopy

After swelling, the fibers were vacuum filtered and examined in a moist state using a Nikon Eclipse Ci-L light microscope equipped with a DS-Fi3 digital camera and a Plan Apo objective lens offering 10× magnification with a numerical aperture of 0.45. This setup was employed to analyze the surface characteristics and fibrillation of the fibers.

#### 2.2.8. Liquid Retention Value (LRV)

The fiber swelling capability was assessed using the Liquid Retention Value (LRV), based on a modified version of the water retention value method as described in ISO 23714:2014. In LRV testing, the wet fiber material is dewatered using a centrifuge (SIGMA Laboratory Centrifuge Type 3-15), which removes the solvents between the fibers, leaving only the liquid retained within the fiber walls and fines. This method provides insight into the solvents’ swelling behavior by evaluating the liquid uptake capacity of the fibers. Mathematically, the LRV is measured as grams of liquid absorbed through swelling per gram of dry pulp after swelling. It is calculated using Equation (1):

(1) $$LRV(g/g)=\frac{m_{wet}-m_{dry}}{m_{dry}}$$

where m_wet_ is the mass of the pulp after centrifugation and before drying, and m_dry_ is the dry weight of the pulp after swelling. As shown in Equation (1), all liquid retention values were normalized to the dry mass after treatment. For NaOH-treated samples, this normalization also compensates for minor carbohydrate losses (<3%) occurring during alkaline exposure. The LRV was measured in duplicates.

#### 2.2.9. Paper Handsheets

The pulps were disintegrated before forming the paper sheets, according to ISO 5263-1:2004. Sheets were then produced using a Rapid-Köthen handsheet former (model RK-4A from PTI Instruments, Oxnard, CA, USA) in accordance with EN ISO 5269-2:2004 [43], aiming for a target grammage of 80 g/m^2^.

#### 2.2.10. Crystallinity Analysis

Sections of the paper sheets from each type of swollen pulp were mounted on a motorized X/Y stage for Wide-Angle X-ray Scattering (WAXS) analysis. This was performed using a laboratory S(W)AXS instrument (SAXSpoint 2.0, Anton Paar, Graz, Austria) equipped with point-focused/slit-collimated Cu-Kα radiation (wavelength of 0.154 nm) and a Dectris EIGER2 R 1 M hybrid pixel area detector (Dectris, Baden-Daettwil, Switzerland). Measurements were conducted under vacuum at 25 °C with a sample-to-detector distance of 150 mm. The 2D WAXS patterns were recorded with a 30-min exposure time and then azimuthally integrated to obtain 1D WAXS curves, displaying scattered intensities relative to the scattering angle (45°). Crystallinity was calculated by fitting a third-order polynomial to the amorphous halo and using five Gaussian curves for peak deconvolution, following established methods [44].

#### 2.2.11. SEC Analysis

Molar mass averages of the used lignins were determined by alkaline SEC analysis (eluent: 10 mM NaOH) using three TSK-Gel columns in series at 40 °C (PW5000, PW4000, PW3000; TOSOH Bioscience, Darmstadt, Germany) with an Agilent 1200 HPLC system (Santa Clara, CA, USA): sample concentration 3 mg/mL, flow rate 1 mL/min and DAD detection at 280 nm. The columns set was calibrated using polystyrene sulfonate reference standards (PSS GmbH, Mainz, Germany). The molar masses at peak maximum (Mp) were 78,400 Da, 33,500 Da, 15,800 Da, 6430 Da, 1670 Da, 891 Da, and 208 Da.

#### 2.2.12. NMR Spectroscopy

For 31P NMR spectroscopy, the lignin samples were dissolved in chloroform/pyridine. N-hydroxy-5-norbinene-2,3-dicarboxylic acid imide (e-HINDI) was used as an internal standard, and chromium (III) acetylacetonate (CR(acac)3) as an NMR relaxation agent. Phosphitylation was done using 3-chloro-4,4,5,5-tetramethyl-1,3,2-dioxaphospholane, and signal attribution was done according to the literature [45,46,47]. Spectra were acquired on a Bruker Avance II 400 (Bruker, Billerica, MA, USA) equipped with a 5 mm N2-cooled cryoprobe head (Prodigy) with z–gradients at room temperature.

#### 2.2.13. Statistical Analysis

Data were evaluated by one-way or two-way analysis of variance (ANOVA [48]) to investigate the significance of the effects of NaOH concentration, ethanol–water ratio, and added lignin concentration on glucose, xylose, mannose, lignin, ash, and LRV. Normality and variance homogeneity were verified [49]. When ANOVA indicated significant effects (*p* < 0.05), pairwise comparisons were performed using Tukey’s HSD post hoc test.

## 3. Results and Discussion

The study was conducted in two parts: first, to evaluate the effectiveness of different swelling agents in influencing PAA fibers, and second, to test these agents as solvents for lignin while combining swelling and impregnation into a single step. Two types of lignin (Kraft and Organosolv) were used.

### 3.1. Part I—Swelling Agents

The pulping parameters used in this study were selected with 90 °C and a processing time of 120 min, ensuring successful pulping, as evidenced by the single fibers illustrated in Figure S2. Two batches of pulp were utilized throughout the entire study to maintain consistency (Figure S1). The pulping process yielded 54% pulp, and the non-dissolved part mainly consisted of knots and residual fiber bundles. However, the non-dissolved part accounted for less than 0.3 wt%. Both pulps were homogeneous and had a similar composition, which contributed to the reliability and reproducibility of the results.

The swelling agents were chosen for their capacity to swell the fibers and to dissolve lignin. In the case of ethanol, lignin solubility peaks around 60–70 wt%, while swelling effects decrease as ethanol content increases (see Section 3.1.2). To cover the range around that solubility peak, ethanol–water mixtures with 50, 70, and 100 wt% ethanol were tested. For sodium hydroxide, mild concentrations of 2 and 4 wt% were selected to avoid mercerization (conversion of cellulose I to cellulose II) and to prevent severe changes to the lignocellulosic composition. Cellulose I has a higher crystalline content and therefore typically better mechanical performance, whereas cellulose II is more chemically accessible for modification or conversion. For reinforcement applications in biocomposites, cellulose I is preferred because it better preserves structural integrity.

#### 3.1.1. Chemical Analysis—Swelling Agents

**Figure 4:**
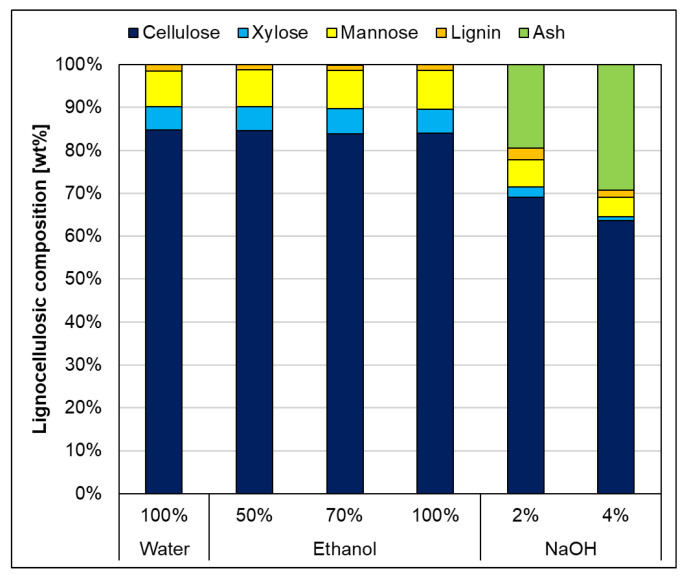
Lignocellulosic composition after swelling with different swelling agents compared to water-swollen fibers as a reference. Standard deviation reported in the Supplementary Materials Table S1.

Looking at the chemical analysis in Figure 4, the lignocellulosic composition showed that treatments with water and aqueous ethanol solutions resulted in fibers with very similar compositions. In contrast, sodium hydroxide treatments led to significant changes: there was a notable increase in ash content, and at a concentration of 2 wt%, a decrease in xylose and mannose content was observed, further decreasing at 4 wt%. This is because sodium hydroxide is known to solubilize lignin and hemicellulose, a property that makes it useful in many pulping techniques. The substantial remaining ash content after sodium hydroxide treatment is most likely caused by the formation of sodium salt complexes with lignin and/or hexuronic acids in the hemicellulose [50]. In addition, the extractives were measured beforehand to ensure extractive-free measurements of the lignocellulosic composition. The extractives were determined gravimetrically but excluded from evaluations (concentrations were below 5 wt%) concerning the impact of solvents and additives on fiber properties, as their inclusion would introduce additional complexity to the interactions among the components.

#### 3.1.2. Liquid Retention Value—Swelling Agents

**Figure 5:**
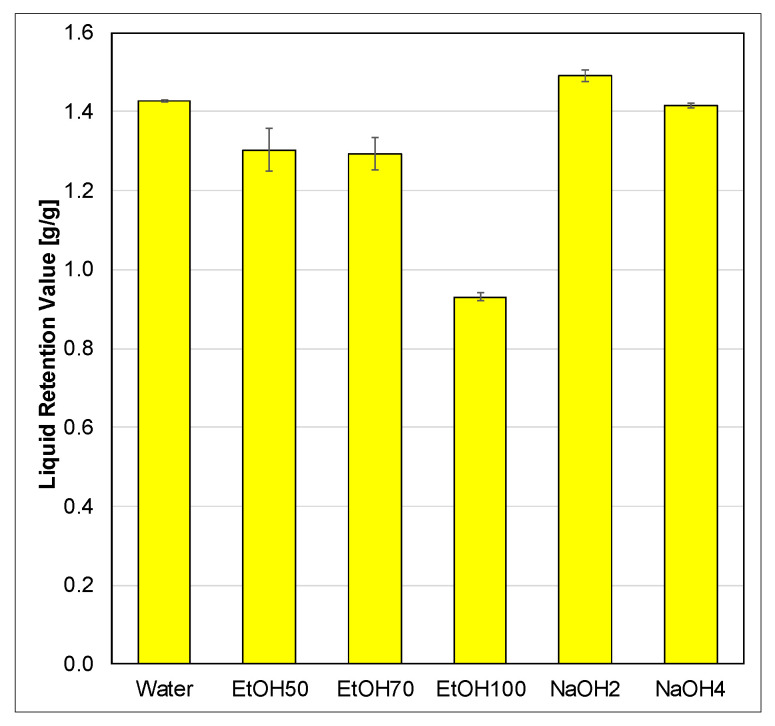
Liquid retention values of different swelling agents.

Swelling experiments were performed for all solvents, including aqueous ethanol solutions and sodium hydroxide aqueous solutions, to investigate their impact on the uptake capacity of the fibers, as shown in Figure 5. The results exhibited relatively small error bars, indicating consistent and reliable measurements. Water served as the reference solvent for swelling, since the fibers were stored moist state with water and demonstrated a similar swelling performance to 4 wt% sodium hydroxide solution (NaOH4). Notably, the 2 wt% sodium hydroxide solution (NaOH2) showed the highest liquid uptake capacity among all solvents tested. However, the values for NaOH-treated samples should ideally be higher [51]. Due to weight loss resulting from polysaccharide dissolution, as evidenced by changes in lignocellulosic composition, the measured values appear lower. The actual phenomenon occurring here is that the lower mass of the pulp results in a thinner, more porous filter pad that is less effective in holding back liquid due to reduced filtration resistance. While this affects practical liquid retention, it does not affect the LRV calculation itself, as it is expressed in relative terms (g/g) based on the input sample mass. However, the weight range specified in the WRV standard (ISO 5263-1:2004) was still met in all tests, ensuring comparability of the liquid retention measurements.

**Figure 6:**
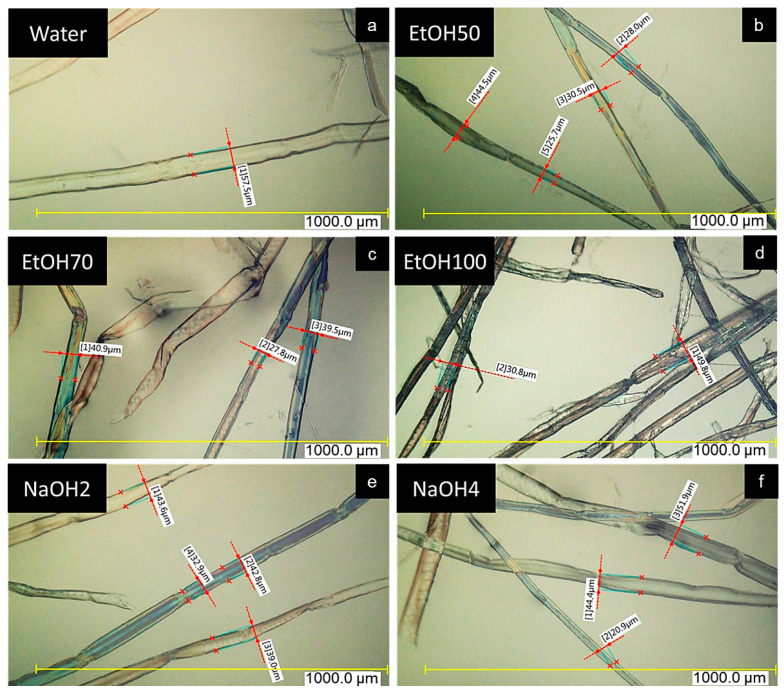
Swollen fibers analyzed in different swelling agents after 4 h: (**a**) water, (**b**) 50% ethanol (EtOH50), (**c**) 70% ethanol (EtOH70), (**d**) 100% ethanol (EtOH100), (**e**) 2% sodium hydroxide (NaOH2), and (**f**) 4% sodium hydroxide (NaOH4).

The ethanol solutions at 50% (EtOH50) and 70% (EtOH70) concentrations yielded similar results, slightly lower than the reference value with water, suggesting a modest reduction in the swelling ability of 9%. In contrast, the 100% ethanol solution (EtOH100) resulted in the lowest liquid uptake capacity, which was confirmed by microscopic images. Figure 6 shows a dried-out and brittle structure in fibers treated with pure ethanol [52].

#### 3.1.3. Light Microscopy—Swelling Agents

Qualitative microscopic examination of the swollen fibers in solution in Figure 6 revealed variations in fiber width and surface morphology, primarily due to the diverse origins of the fibers, heartwood, sapwood, and juvenile wood, which made it challenging to draw definitive conclusions regarding their swelling abilities from optical measurements. In the EtOH50, the fibers exhibited visible neckings along their lengths but remained evenly swollen overall. When treated with EtOH70, the fibers showed more pronounced neckings and wrinkles on their surfaces, giving them a dried-out appearance. The fibers immersed in EtOH100 appeared significantly dried out, with sharp neckings along their lengths, a wrinkled surface, and uneven widths. In contrast, fibers treated with NaOH2 showed even swelling with small neckings and a smooth surface, a structure similar to that observed in NaOH4. Fibers swollen in water exhibited characteristics comparable to those treated with NaOH solutions, showing even swelling and smooth surfaces.

#### 3.1.4. Crystallinity

**Figure 7:**
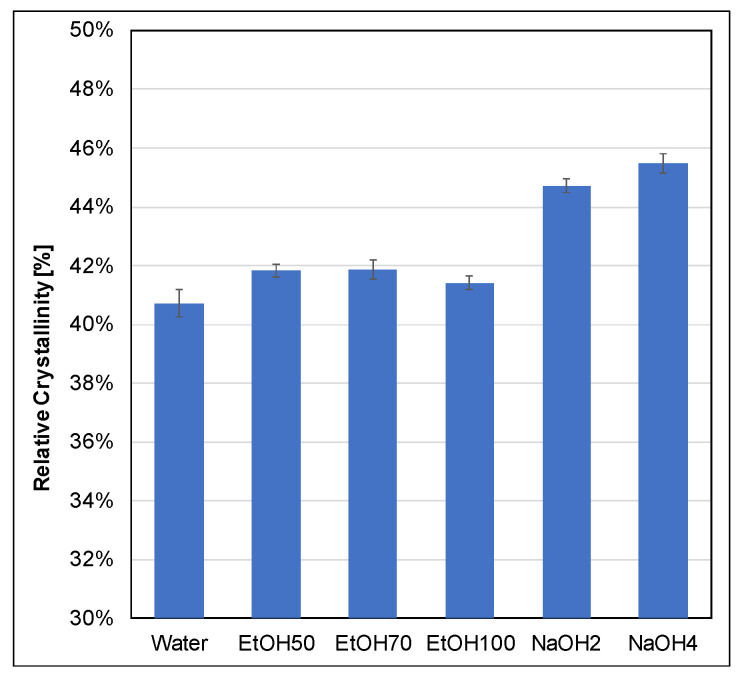
Relative crystallinity content after swelling with different swelling agents.

**Figure 8:**
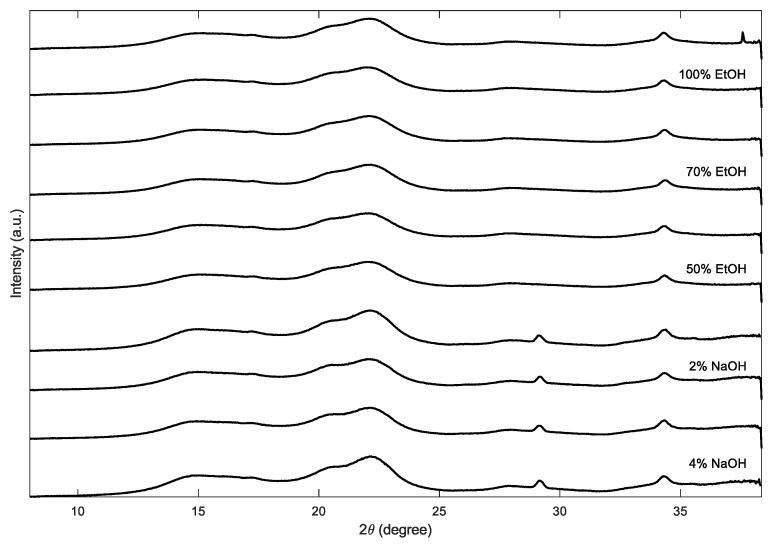
Stacked wide-angle X-ray diffraction (WAXD) patterns (2θ, °) of cellulose samples after solvent/alkali treatments. Traces are shown as replicate pairs in the following top-to-bottom order: 100% EtOH, 70% EtOH, 50% EtOH, 2% NaOH, 4% NaOH. For clarity, data below 8° 2θ were excluded, and each spectrum was min–max normalized on its cropped range to 0–100 arbitrary units before stacking.

During sheet formation, lignin would be washed out to a certain extent, compromising consistency. As previously shown in Figure 4, treatments with NaOH resulted in a notable reduction in polysaccharide content compared to ethanol-based mixtures. This observation is further supported by Figure 7, which shows an increase in the crystalline fraction for both NaOH concentrations. NaOH solvents preferentially remove hemicellulose and amorphous cellulose regions, preserving the crystalline fraction and thus increasing the measured crystallinity compared to the water-treated reference. As expected, ethanol–water mixtures led to only slight increases in crystallinity, with lower ethanol concentrations showing relatively higher values—likely due to subtle solvent–fiber interactions favoring cellulose chain rearrangement [53]. These findings are supported by WAXD data provided in the Supplementary Materials. A small peak near 2θ ≈ 29° was observed in the diffractograms of the NaOH-treated samples, most likely resulting from residual sodium salts. However, this artifact does not significantly influence the calculated crystallinity, as confirmed by comparative analysis of the WAXD curves (Figure 8). Crystallinity was only measured for the swelling agents without additional lignin because it was not possible to form paper sheets with the impregnated fibers.

### 3.2. Part II—Impregnation

**Figure 9:**
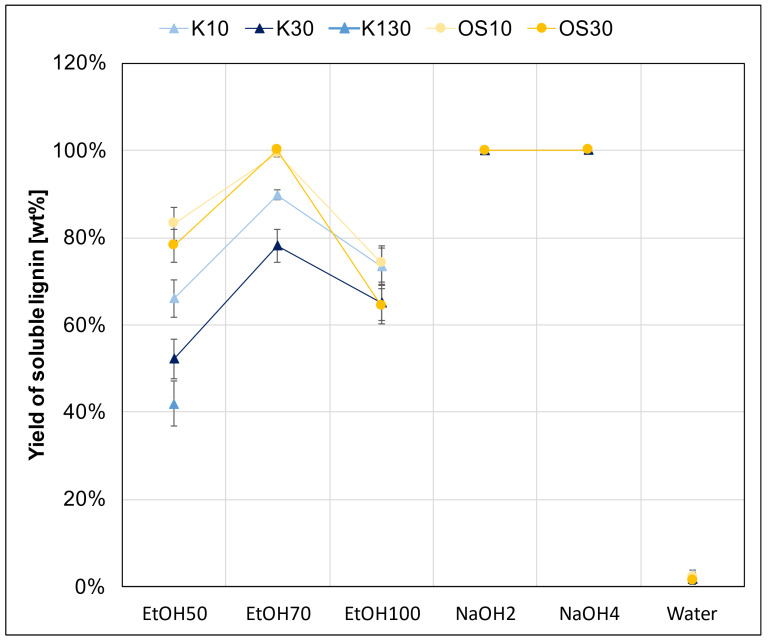
Solubility of lignins (Kraft and Organosolv) in the swelling agents.

This second part aimed to test the swelling agents as solvents for lignin while combining swelling and impregnation into a single step. The solubility of lignin in the different swelling agents was analyzed, and the resulting supernatants were diluted to the required concentrations for further processing. As illustrated in Figure 9, the swelling agent “water” was not suitable to dissolve lignin due to its hydrophobic properties. Consequently, it was excluded from further experiments.

Both lignins showed the highest solubility in NaOH. Within the ethanol mixtures, EtOH70 showed very similar solubility to NaOH for OS10 and OS30. In the case of the Kraft lignins, K10 and K30, they also had the highest ethanol solubility with EtHO70, but it was lower than that of the Organosolv (OS) lignin. Both lignins showed a similar trend through the ethanol mixtures. These findings align very well with the literature, as ethanol–water mixtures show a peak in solubility around 60–70 wt% of ethanol [34].

The soluble lignin yield was measured once as an initial test with the double initial concentration (20 and 60 g/L) to determine the appropriate starting concentration needed to achieve the target supernatant concentration during the impregnation step, as shown in Table 1. Lignin concentrations of 10 and 30 g/L were selected to ensure sufficient solubility in each swelling agent and enhance homogeneous distribution within the fiber structure. Lower concentrations like these help reduce the risk of premature precipitation and support more homogeneous distribution. To evaluate the maximum lignin uptake potential under favorable solubility conditions, an elevated concentration of 130 g/L Kraft lignin supernatant (K130) with the solvent EtOH50. Ethanol–water systems are known to provide good solubility for hydrophobic compounds like lignin, while maintaining moderate swelling behavior in natural fibers. The 50% ethanol composition was selected as a compromise between swelling efficiency and lignin solubility, enabling the system to act both as a swelling agent and as a carrier solvent. This high-concentration point serves to test the saturation threshold of the impregnation process and to investigate whether increasing the lignin load improves fiber uptake beyond the levels observed at 10 or 30 g/L. As expected, K130 resulted in a lower solubility yield compared to supernatants prepared from lower initial concentrations [34,54].

#### 3.2.1. Chemical Analysis—Impregnation

**Figure 10:**
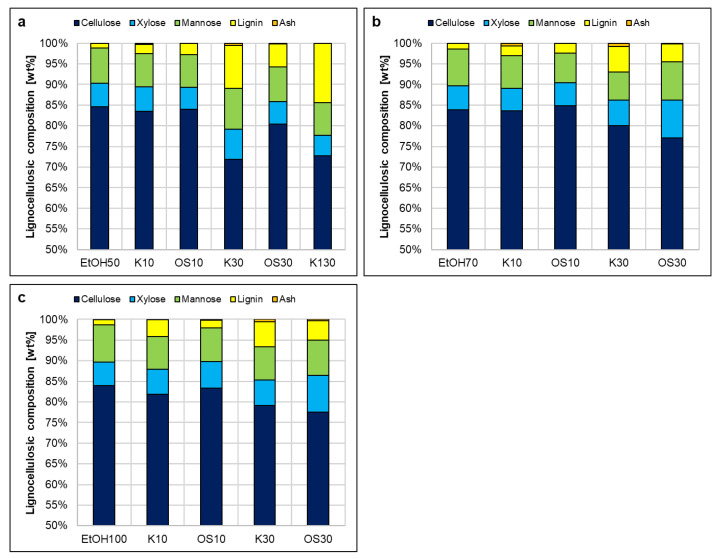
Lignocellulosic composition of pulp after impregnation in solvent agents (**a**) EtOH50, (**b**) EtOH70, and (**c**) EtOH100. Leftmost columns are the blank (pure solvent), 10/30 refers to 10 g/L or 30 g/L lignin concentration in the solvent, K is Kraft lignin, and OS is organosolv lignin. Standard deviation reported in the Supplementary Materials (Table S2).

The fibers were impregnated with lignin solutions (supernatants) prepared from different swelling agents. Lignin was soluble in all swelling agents at the targeted concentrations of 10 g/L and 30 g/L, except for water. Figure 10a,b illustrate similar trends in lignocellulosic composition after swelling in ethanol mixtures EtOH50 and EtOH70. Retained lignin for K10 and OS10 ranged between 2 and 3 wt%, while OS30 was retained at around 5 wt%. At the same time, K30 showed significantly higher lignin impregnation value (LIV) (>10 wt%) with EtOH50 compared to EtOH70 (~7 wt%) (Figure 10a). In Figure 10c, EtOH100 resulted in higher LIV for both K10 and K30 compared to Organosolv lignin. OS30 showed consistent retention across all ethanol mixtures, whereas K10 had the highest, and OS10 the lowest, retention in EtOH100. Ash content remained negligible, and polysaccharide composition was largely unaffected. Overall, EtOH50 led to the highest LIV for all concentrations, with the exception of one outlier (K10/EtOH100). In contrast, increasing the concentration to 130 g/L (K130) in EtOH50 did not result in a significant increase in LIV compared to K30, suggesting that lignin uptake by the fibers is not directly proportional to its concentration in the impregnation solution.

**Figure 11:**
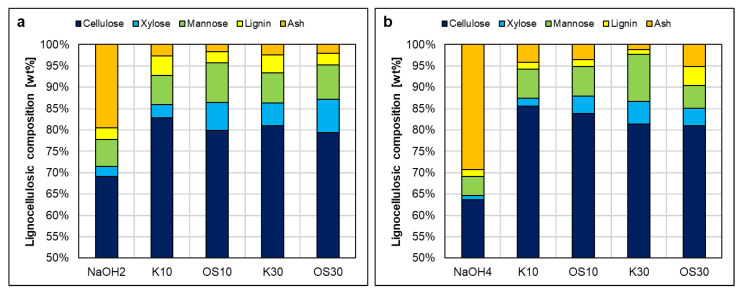
Lignocellulosic composition of pulp after impregnation in solvent agents (**a**) NaOH2 and (**b**) NaOH4. Leftmost columns are the blank (pure solvent), 10/30 refers to 10 g/L or 30 g/L lignin concentration in the solvent, K is Kraft lignin, and OS is organosolv lignin. Standard deviation reported in the Supplementary Materials (Table S2).

Sodium hydroxide, on the other hand, had an effect on the content of the polysaccharides, particularly reducing hemicellulose, when considering the cellulose/hemicellulose ratio. After swelling with sodium hydroxide, the ratio increased from approximately 6:1 (initial pulp) to 8:1 for NaOH2 and to 12:1 for NaOH4. When lignin was involved, the rate was much lower. Figure 10 shows higher ash content in fibers swollen with NaOH2 and NaOH4 compared to ethanol–water mixtures, primarily due to residual sodium salts from the swelling with sodium hydroxide. However, the presence of lignin significantly reduced ash content, possibly because the precipitation of lignin on the fibers reduces the available surface for sodium salts to attach, preventing sodium salt accumulation. In Figure 11a, Kraft lignin showed significantly higher retention than Organosolv lignin, whereas Figure 11b shows similar retention values for both Kraft concentrations (K10, K30) and OS10. Interestingly, OS30 in NaOH4 was retained nearly as effectively as K30 in NaOH2. Although NaOH dissolves both lignin types, Kraft lignin impregnation outperforms Organosolv lignin, suggesting that solubility alone does not directly correlate with impregnation efficiency. The superior retention of Kraft lignin may result from structural or particle size differences, enabling more effective precipitation onto the fiber surface compared to Organosolv lignin. However, a limitation of this study is the insufficient investigation of precipitation timing and mechanisms, which are critical for fully understanding and optimizing impregnation efficiency.

#### 3.2.2. Liquid Retention Value—Impregnation

**Figure 12:**
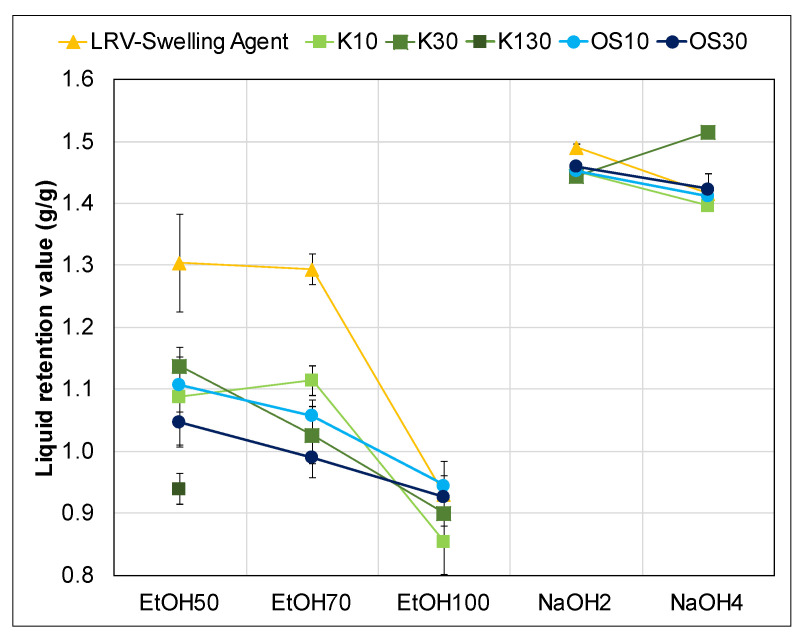
Liquid retention value of different solvents and their supernatants with three lignin concentrations (10 g/L and 30 g/L, 130 g/L).

Figure 12 illustrates the impregnated lignin value after a combined 4-h swelling and impregnation treatment. Comparing fibers treated with pure solvents to those treated with lignin-containing supernatants, a notable decrease in LRV was observed for EtOH50 and EtOH70. In contrast, LRV for EtOH100, NaOH2, and NaOH4 remained largely unchanged between the pure solvents and their corresponding supernatants, with the exception of K30 in NaOH4, which showed a slight increase in LRV.

The lowest LRV was recorded for K130, consistent with the trend observed for K30 and OS30, where higher lignin concentrations led to reduced LRV. Although the differences in LRV among supernatants were relatively small due to larger error margins, the overall trend suggests that the presence of lignin reduces LRV in ethanol-based systems, particularly in EtOH50 and EtOH70. EtOH100, however, consistently yielded low LRV across all conditions, making differentiations difficult. Additionally, the variability in LRV was more extensive for the ethanol solutions than for the NaOH solutions, suggesting a more severe effect of supernatant on the swelling behavior in aqueous ethanol solutions.

The relationship between fiber swelling and lignin impregnation efficiency was studied to determine if increased fiber swelling improves binder uptake. In theory, swelling expands the internal spaces within fibers, potentially enhancing binder adhesion. Liquid retention measurements supported this concept, as higher LRVs were observed in fibers treated with swelling agents that induced greater expansion. However, comparing the data in Figure 10 and Figure 11 with the LRV in Figure 12 showed no direct correlation between the extent of swelling and the LIV. This unexpected result indicates that impregnation efficiency is influenced by more than just physical expansion. Compositional factors, such as xylan and glucomannan in the fiber matrix, likely play a significant role in governing binder interaction and deposition.

#### 3.2.3. Lignin Impregnation Value

**Figure 13:**
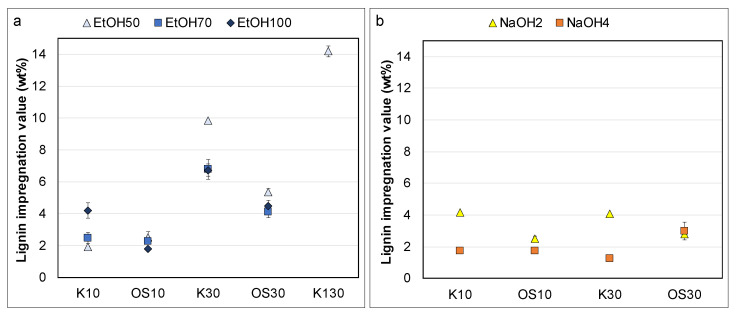
Impregnated lignin content after swelling coupled with impregnation for (**a**) ethanol mixtures and (**b**) aqueous sodium hydroxide solutions.

The lignin content in the impregnated fibers was determined in the centrifugated and dried pads from the LRV measurement, using the Klason method. The results are shown in Figure 13a,b, where Kraft lignin showed significantly higher lignin content at the lower concentration (K10) for EtOH100 and NaOH2 (4.18 wt% and 4.14 wt%) compared to O10 with the same solvents (1.89 wt% and 2.50 wt%). For NaOH4, K10 and O10 showed similar values (1.72 wt% and 1.73 wt%), like at EtOH50, where the difference was minimal. However, at higher lignin concentrations, the trend changes significantly. For K30 and O30, the LIV in all EtOH mixtures was notably higher compared to both NaOH concentrations, where the measured content was considerably lower.

Increasing the lignin concentration in the supernatant generally led to greater lignin uptake by the fibers, a trend consistently observed across both tested lignin types. However, this trend was not observed under sodium hydroxide conditions, where lignin contents remained similar at both 10 and 30 g/L concentrations. Additionally, although NaOH-treated samples exhibited higher LRV compared to ethanol-treated ones, they generally showed lower lignin contents, except for the K10 sample treated with NaOH2. This discrepancy suggests that lignin might remain fully dissolved or chemically react under NaOH conditions, thus reducing its effective availability for fiber impregnation. The lack of a direct correlation between LRV and lignin uptake also indicates that hemicellulose-mediated hydrogen bonding and lignin structuration exert a greater influence on lignin attachment than physical swelling alone.

In this context, hemicelluloses likely play a crucial role in enhancing fiber–binder interactions. Positioned on the cellulose fibril surfaces, hemicelluloses naturally bridge cellulose and lignin, significantly improving interfacial adhesion. Their capacity to form hydrogen bonds with cellulose and to interact with lignin contributes to a more cohesive and integrated fiber network. Conversely, NaOH treatment also solubilizes hemicellulose, as confirmed by the analysis in Figure 6. Subtle variations observed in the WAXD signals of NaOH-treated samples further support this hypothesis, suggesting possible structural modifications or altered interactions between lignin and the alkali-modified fiber surfaces.

This discrepancy suggests that lignin may stay completely dissolved or chemically react in NaOH environments, reducing its effective availability for fiber impregnation. In this context, hemicelluloses likely play an important role in enhancing fiber–binder interactions. Positioned on the surface of cellulose fibrils, they serve as natural linkers between cellulose and lignin, contributing to interfacial adhesion. Their ability to form hydrogen bonds with cellulose and interact with lignin supports a more cohesive fiber network. However, NaOH also solubilizes hemicellulose, which is confirmed by the analysis in Figure 4. Subtle variations in the WAXD signals of NaOH-treated samples support this hypothesis, indicating possible structural changes or interactions between lignin and alkali-modified fiber surfaces.

Swelling and lignin impregnation proceed concurrently under the applied conditions. Precipitation timing appeared to be a critical factor affecting LIV. The solvent composition and water content determine the onset and rate of lignin precipitation via solvent shifting. When wet fibers are introduced into ethanol–water mixtures, the increased water content in the supernatant solution triggers immediate lignin precipitation, a process known as solvent shifting, which could explain the outlier K10 in EtOH100 (Figure 13). This immediate precipitation results in partial lignin deposition during the swelling and impregnation phases. The solvent shift phenomenon is well-documented in the literature, where water is commonly used as an anti-solvent to fractionate lignin via controlled precipitation [55]. Time-resolved tests indicated that both liquid uptake and lignin adsorption stabilized within approximately 4 h, suggesting that diffusion and aggregation kinetics, rather than swelling alone, govern the overall process.

Statistical evaluation across NaOH- and EtOH-based swelling systems revealed that the NaOH concentration is the predominant factor of fiber composition and swelling behavior. Increasing NaOH from 2 to 4 wt% significantly enhanced the relative glucose content and water retention while noticeably reducing lignin, confirming effective delignification and partial hemicellulose removal. EtOH–water mixtures, in contrast, primarily influenced lignin deposition and liquid retention, where intermediate mixtures (50–70 wt%) yielded higher residual lignin and ash, whereas pure ethanol improved carbohydrate recovery but reduced fiber swelling. The added lignin concentration showed no independent main effect; however, its interaction with solvent composition modified both glucose and LRV.

#### 3.2.4. Microscopy—Impregnation

**Figure 14:**
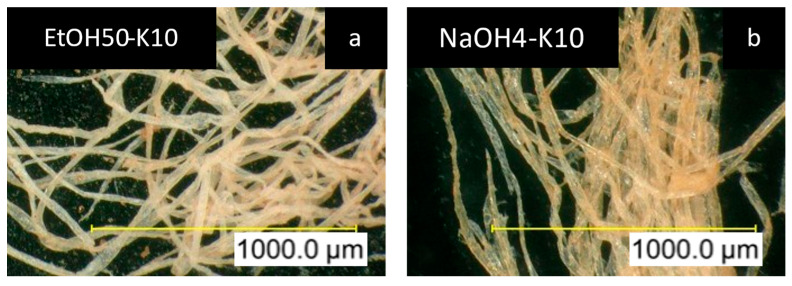
**(a**) Microscopic pictures of impregnated holocellulose fibers in ethanol–water mixtures and (**b**) sodium hydroxide.

Figure 14a depicts the above-mentioned solvent shift for EtOH50 with K10, which shows the precipitation of lignin in the form of particles on the fibers and a slight change in color of the fibers to light brown. In comparison with Figure 14b, K10 in NaOH4 shows a weak coverage of a lignin film on the fibers, and the sodium salts form salt crystals around the fiber. All other microscopic pictures show a similar pattern, regardless of the lignin type or concentration. These pictures are provided in the SI, Figures S1 and S2.

Additionally, the solubility of lignin in various solvents was examined to assess its impact on impregnation efficiency. Results indicated that higher lignin solubility is negatively correlated with impregnation effectiveness, as illustrated in Figure 9 and Figure 13. This is likely because highly soluble lignin remains dissolved longer, precipitating too late for effective deposition onto fibers. Furthermore, such lignin particles may be too small or washed away during subsequent processing steps. Additionally, a staged addition of water to ethanol-rich supernatants could act as a controlled antisolvent step, triggering in-fiber lignin precipitation and thereby improving deposition efficiency. Such solvent-shifting strategies may represent a viable route to further enhance lignin incorporation.

## 4. Conclusions

This study demonstrates a single-step approach that combines fiber swelling and lignin impregnation using holocellulose fibers. Among the investigated solvent systems, NaOH 2 wt% produced the highest swelling (LRV ≈ 1.4 g/g), while EtOH 50 wt% achieved the most effective lignin uptake (LIV ≈ 10 wt% for Kraft lignin). Interestingly, lignin impregnation efficiency was not directly correlated with the degree of fiber swelling. Although greater swelling theoretically provides more internal space for binder penetration, no consistent relationship between swelling extent and lignin uptake was observed. Instead, impregnation efficiency appears to be governed primarily by solvent composition, lignin solubility, and precipitation dynamics.

A key observation was that higher lignin solubility in the impregnation solvent reduced deposition efficiency, as lignin remained dissolved and precipitated too late or incompletely on the fiber surface. In contrast, introducing water-swollen fibers into ethanol-rich solutions triggered partial and immediate lignin precipitation through solvent shifting, which promoted more effective lignin deposition. These findings underscore the decisive role of precipitation timing and solvent–binder interactions in achieving uniform impregnation. NaOH primarily drives chemical transformation, ethanol controls physical accessibility, and lignin addition modulates the interaction between these effects, collectively establishing NaOH as the most statistically influential treatment factor.

Overall, the results confirm the feasibility of a one-step preconditioning process that integrates swelling and impregnation, providing new insight into the interplay between fiber structure, solvent properties, and lignin behavior. Lignin deposition is influenced not only by the accessible surface area generated during swelling but also by aggregation behavior and solution chemistry. To evaluate these mechanisms further, future research should include kinetic studies of lignin precipitation, the influence of lignin chemical structure on deposition behavior, and quantitative surface-area and surface-energy analyses. Moreover, controlling solvent-shifting effects and evaluating the mechanical performance of pre-impregnated composites will be essential for developing scalable and sustainable manufacturing processes for lignin-bonded biocomposites.

## Figures and Tables

**Table 1:** Concentration of Kraft and Organosolv lignin supernatants used for fiber impregnation.

Solvent	Supernatant Concentration				
	K10	K30	K130	OS10	OS30
	g/L	g/L	g/L	g/L	g/L
EtOH50	9.88	30.00 ^b^	130.00 ^b^	9.74	30.00 ^b^
EtOH70	9.14	30.00 ^b^		10.09	29.21
EtOH100	9.45	30.00 ^b^		9.55	30.00 ^b^
NaOH2 ^a^	10.00	30.00		10.00	30.00
NaOH4 ^a^	10.00	30.00		10.00	30.00

^a^ Assumption that all lignin was dissolved. ^b^ Assumption of concentration after calculated dilution.

## Data Availability

The original contributions presented in this study are included in the article/Supplementary Material. Further inquiries can be directed to the corresponding author.

## Supplementary Materials

The following supporting information can be downloaded at: https://www.mdpi.com/article/10.3390/polym17233103/s1. Figure S1: Pulp yield of the two batches used in this study; Figure S2: Single fibers after pulping (90 °C and 120 min); Figure S3: Microscopic pictures of the impregnated fibers in different swelling agents (ethanol 50, 70, and 100 wt%; sodium hydroxide 2 and 4 wt%) with a lignin concentration of 10 g/L (two different lignins; K-Kraft and OS-Organosolv); Figure S4: Microscopic pictures of the impregnated fibers in different swelling agents (ethanol 50, 70, and 100 wt%; sodium hydroxide 2 and 4 wt%) with a lignin concentration of 30 g/L (two different lignins; K-Kraft and OS-Organosolv); Figure S5: WAXS diffraction intensity signal over 2θ (1D) for PAA-treated samples at 90 °C and 120 min. The evaluation of the signal includes a third-order polynomial fit for the amorphous halo and a Gaussian fit for the crystalline contribution; Table S1: Lignocellulosic composition after swelling with different swelling agents; Table S2: Average and deviation of the lignocellulosic composition after impregnation.
