# Supplementary material for: Effect of Different Aqueous Solvents with and Without Solubilized Lignin on the Swelling Behavior of Holocellulose Fibers
Source: Polymers (Basel). 2025 Nov 22;17(23):3103. doi: 10.3390/polym17233103 (PMC12694391; doi:10.3390/polym17233103)
Supplement: Supplementary file 1 [file polymers-17-03103-s001.zip › polymers-3964997-supplementary.pdf]

Supplementary Information

for

Tailored holocellulose fibers from spruce wood chips: Optimizing peracetic acid pulping conditions

Cornelia Hofbauer; Thomas Harter; Ulrich Hirn; Michael Harasek; Luis Zelaya-Lainez; Josef Füssl; Markus Lukacevic; Sebastian Serna Loaiza

Corresponding author:

Christian Doppler Laboratory for Next-Generation Wood-Based Biocomposite, Institute of Chemical, Environmental and Bioscience Engineering, TU Wien, Vienna, Austria

E-mail: cornelia.hofbauer@tuwien.ac.at

Pulping

Initial holocellulose batch for swelling, process parameters 90 °C and 120 min reaction time.

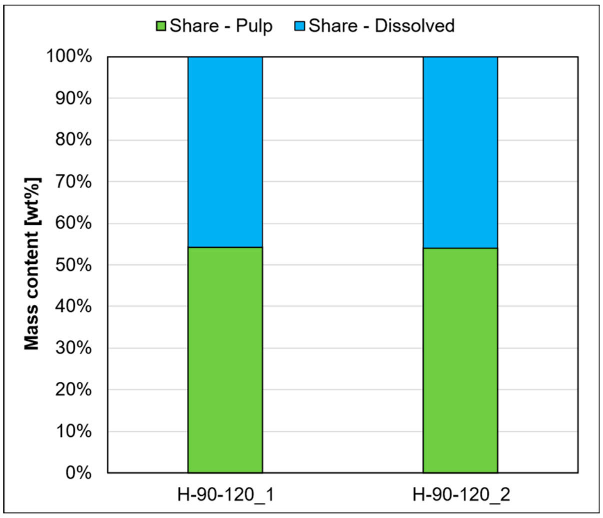

Figure S1 Pulp yield of the two batches used in this study

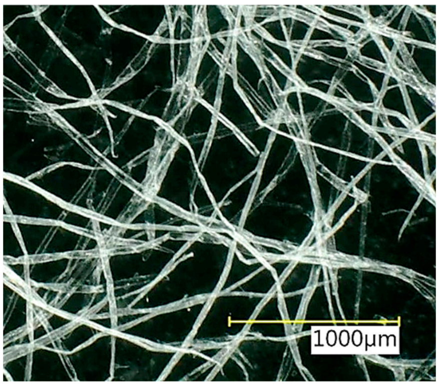

Figure S2 Single fibers after pulping (90°C and 120min)

Swelling

Lignocellulosic composition after swelling without lignin impregnation

Table S1 Lignocellulosic composition after swelling with different swelling agents

Sample Code	Cellulose/Glucose		Hemicellulose		Lignin		Ash	
	Average	Deviation	Average	Deviation	Average	Deviation	Average	Deviation
	wt%	wt%	wt%	wt%	wt%	wt%	wt%	wt%
Water	89.7	0.56	14.44	0.52	1.53	0.12	0.06	0.05
EtOH50	82.9	0.39	14.02	0.77	1.07	0.13	0.03	0.03
EtOH70	81.07	0.42	14.25	0.81	1.24	0.23	0.07	0.12
EtOH100	81.88	0.70	14.37	0.67	1.25	0.30	0.01	0.03
NaOH2	68.64	0.22	8.54	0.24	2.70	0.02	19.37	0.31
NaOH4	65.47	0.31	5.55	0.30	1.68	0.06	30.16	0.41

Impregnation

Table S2 Average and deviation of the lignocellulosic composition after impregnation

Sample Code	Cellulose/Glucose		Hemicellulose		Lignin		Ash	
	Average	Deviation	Average	Deviation	Average	Deviation	Average	Deviation
	wt%	wt%	wt%	wt%	wt%	wt%	wt%	wt%
EtOH50_K10	83.48	0.72	14.00	0.90	2.23	0.15	0.29	0.04
EtOH70_K10	83.62	0.71	13.32	0.78	2.45	0.21	0.61	0.27
EtOH100_K10	81.87	0.94	13.95	0.71	4.05	0.25	0.12	0.02
NaOH2_K10	82.89	0.50	9.85	0.27	4.53	0.05	2.72	0.28
NaOH4_K10	85.58	0.37	8.68	0.06	1.64	0.08	4.10	0.36
EtOH50_OS10	83.94	0.30	13.30	0.25	2.70	0.06	0.06	0.00
EtOH70_OS10	84.85	0.07	12.70	0.08	2.41	0.16	0.03	0.00
EtOH100_OS10	83.39	0.24	14.57	0.30	1.83	0.08	0.20	0.02
NaOH2_OS10	79.85	0.58	15.87	0.40	2.55	0.55	1.73	0.44
NaOH4_OS10	83.87	0.74	10.94	1.26	1.65	0.16	3.54	0.37
EtOH50_K30	71.86	0.99	17.26	0.94	10.36	0.00	0.52	0.06
EtOH70_K30	80.07	6.08	12.98	5.20	6.16	0.82	0.79	0.06
EtOH100_K30	79.11	2.00	14.24	1.27	6.11	0.63	0.54	0.10
NaOH2_K30	80.93	0.58	12.47	1.13	4.18	0.28	2.42	0.27
NaOH4_K30	81.33	6.44	16.32	6.46	1.14	0.22	1.21	0.20
EtOH50_OS30	80.35	0.07	13.89	0.05	5.57	0.01	0.19	0.03
EtOH70_OS30	77.10	0.15	18.36	0.06	4.41	0.32	0.13	0.12
EtOH100_OS30	77.50	1.61	17.42	1.06	4.70	0.56	0.38	0.01
NaOH2_OS30	79.37	0.42	15.80	0.01	2.78	0.18	2.04	0.25
NaOH4_OS30	80.94	4.43	9.39	0.93	4.48	1.09	5.19	2.41
EtOH50_K130	71.58	1.14	13.31	1.22	14.17	0.01	0.94	0.06

Low lignin concentration of 10 g/l, dissolved in different swelling agents

	K10	OS10
EtOH50	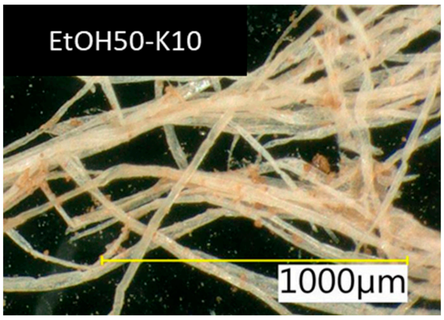	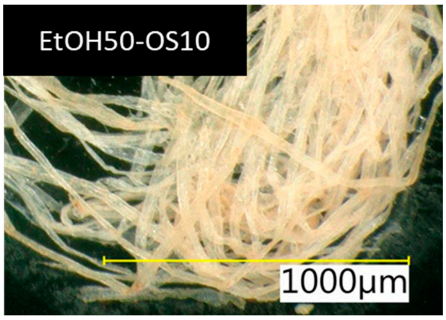
EtOH70	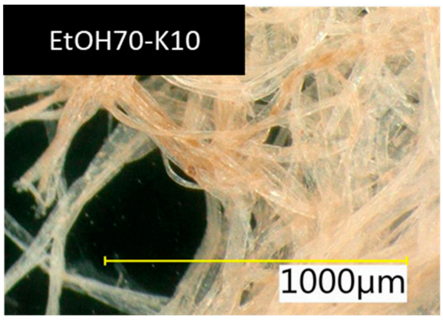	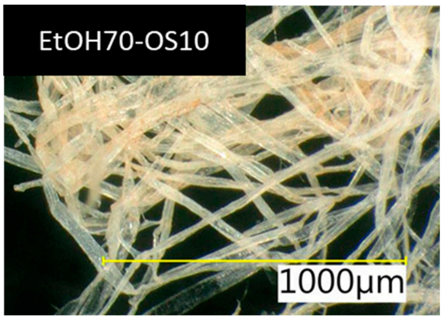
EtOH100	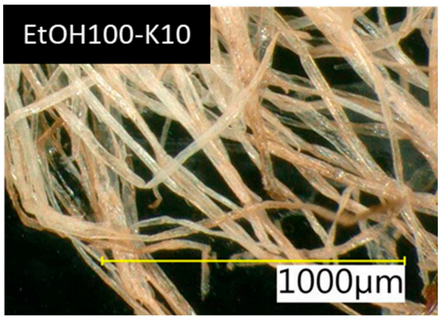	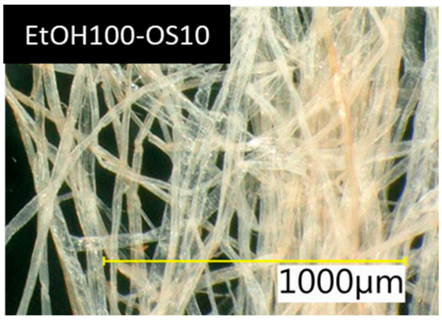
NaOH2	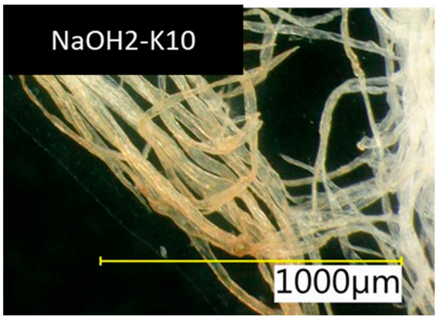	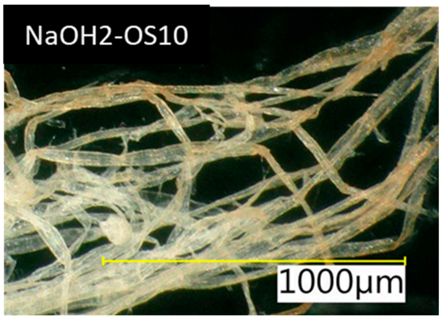
NaOH4	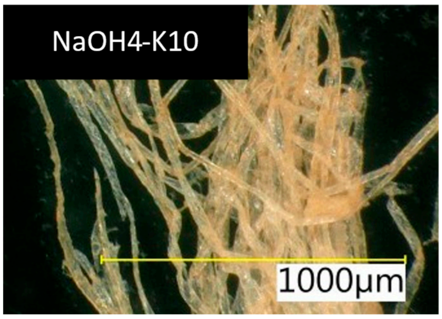	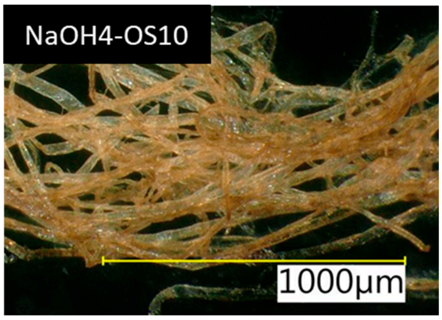

Figure S3: Microscopic pictures of the impregnated fibers in different swelling agents (ethanol 50, 70, and 100 wt%; sodium hydroxide 2 and 4 wt%) with a lignin concentration of 10 g/l (two different lignins; K-Kraft and OS-Organosolv)

High lignin concentration of 30 g/l, dissolved in different swelling agents

	K30	OS30
EtOH50	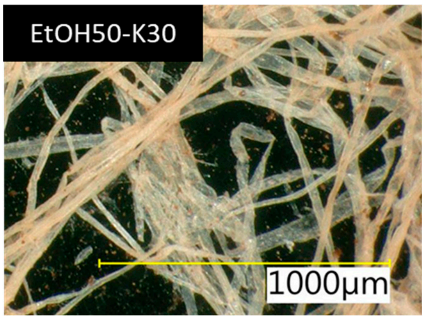	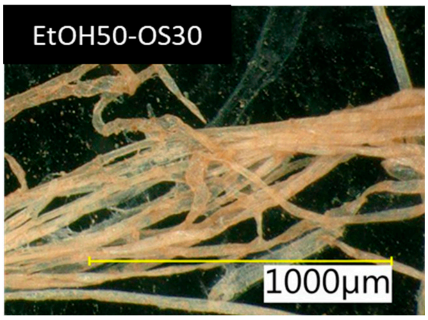
EtOH70	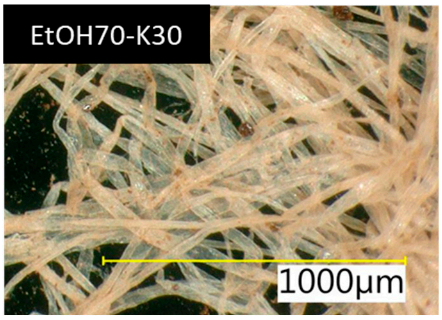	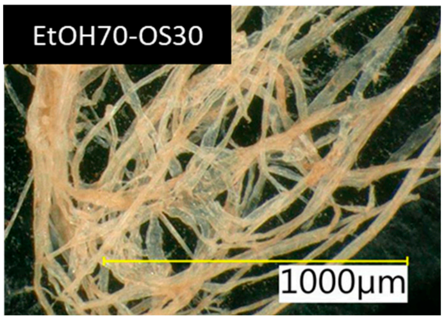
EtOH100	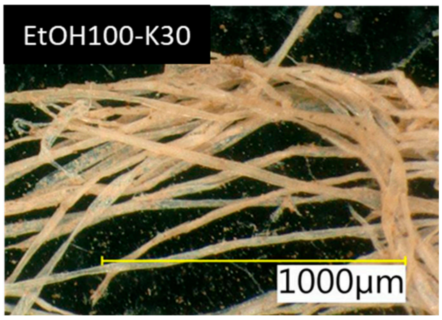	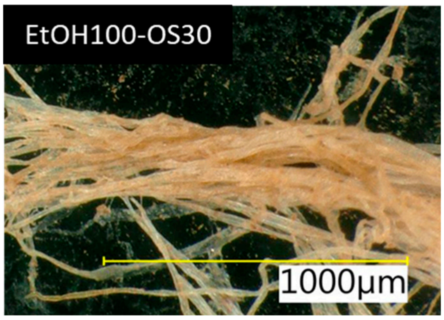
NaOH2	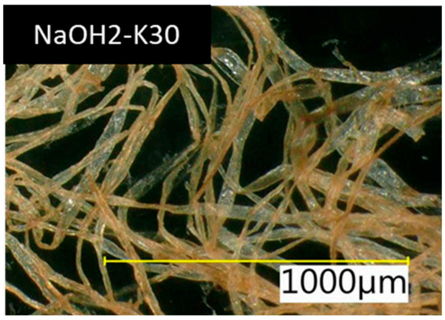	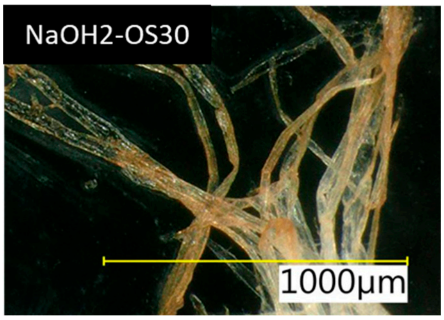
NaOH4	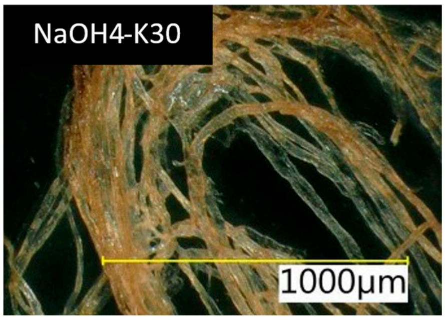	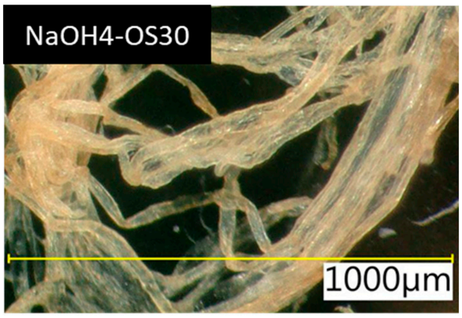

Figure S4: Microscopic pictures of the impregnated fibers in different swelling agents (ethanol 50, 70, and 100 wt%; sodium hydroxide 2 and 4 wt%) with a lignin concentration of 30 g/l (two different lignins; K-Kraft and OS-Organosolv)

Crystallinity

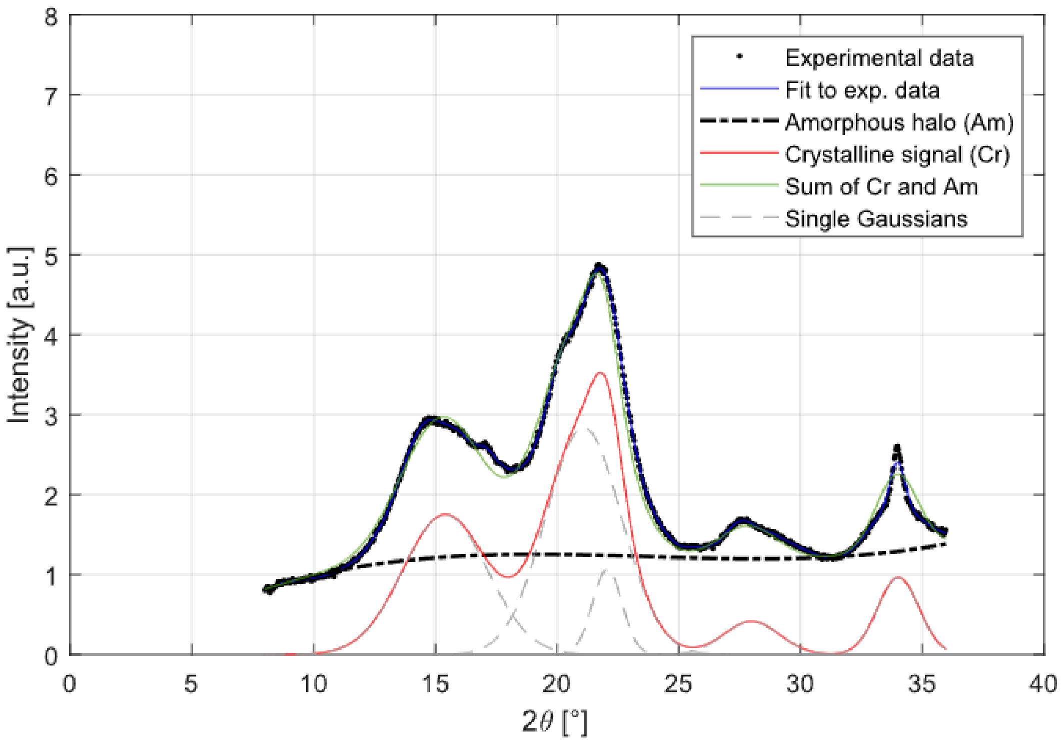

Figure S5: WAXS diffraction intensity signal over 2θ (1D) for PAA-treated samples at 90°C and 120min. The evaluation of the signal includes a third-order polynomial fit for the amorphous halo and a Gaussian fit for the crystalline contribution
